# Bi-allelic loss of function variants in SLC30A5 as cause of perinatal lethal cardiomyopathy

**DOI:** 10.1038/s41431-020-00803-8

**Published:** 2021-02-05

**Authors:** Johann Kaspar Lieberwirth, Pascal Joset, Anja Heinze, Julia Hentschel, Anja Stein, Antonella Iannaccone, Katharina Steindl, Alma Kuechler, Rami Abou Jamra

**Affiliations:** 1 Institute of Human Genetics, University Medical Center Leipzig, Leipzig, Germany; 2 Institute of Medical Genetics, University of Zurich, Schlieren, Switzerland; 3 Department of Pediatrics I, Division of Neonatology, University Medical Center Essen, University Duisburg—Essen, Essen, Germany; 4 Department of Gynecology and Obstetrics, University Medical Center Essen, University Duisburg—Essen, Essen, Germany; 5 Institute of Human Genetics, University Medical Center Essen, University of Duisburg—Essen, Essen, Germany

**Keywords:** Cardiovascular diseases, Development, Medical genetics, Medical genomics

## Abstract

Perinatal mortality is a heavy burden for both affected parents and physicians. However, the underlying genetic causes have not been sufficiently investigated and most cases remain without diagnosis. This impedes appropriate counseling or therapy. We describe four affected children of two unrelated families with cardiomyopathy, hydrops fetalis, or cystic hygroma that all deceased perinatally. In the four patients, we found the following homozygous loss of function (LoF) variants in *SLC30A5* NM_022902.4:c.832_836del p.(Ile278Phefs*33) and NM_022902.4:c.1981_1982del p.(His661Tyrfs*10). Knockout of *SLC30A5* has previously been shown a cardiac phenotype in mouse models and no homozygous LoF variants in *SLC30A5* are currently described in gnomAD. Taken together, we present *SLC30A5* as a new gene for a severe and perinatally lethal form of cardiomyopathy.

## Introduction

Stillbirth occurs in developed countries with a frequency of 3–4 per 1000 pregnancies [1–3]. The possible causes of stillbirths are very heterogeneous and include, but are not limited to infections, maternal risk factors (e.g., diabetes, obesity, smoking), pregnancy complications such as placenta dysfunction, preeclampsia, or asphyxia, congenital fetal anomalies, and genetic conditions [4]. Especially for genetic causes, the underlying mechanisms have not yet been sufficiently understood. In developed countries, hydrops fetalis occurs in ~9% of all stillbirths [5] with a general frequency of around 1 out of 2500 pregnancies [6, 7]. The majority (90%) of cases of fetal hydrops in western countries have shown to be non-immunological (NIHF), while cardiovascular causes (20%) represent the most frequent origin of this condition [6, 8]. Apart from chromosomal disorders and classical syndromes such as Noonan’s syndrome, underlying causes of fetal hydrops are difficult to decipher. Still only half of these cases can be explained [9] and numerous underlying genes remain to be explored. Only with the advent of broad-based in-depth genetic analyses including exome sequencing (ES), an even greater variety of genetic causes for fetal anomalies can be detected [10, 11]. Identifying the precise etiology of NIHF is essential for effective clinical management of these pregnancies as well as for better counseling of families regarding prognosis, risk of recurrence, and prenatal diagnostics [6].

Here we describe four individuals from two unrelated families in which we identified homozygous loss of function (LoF) variants in the zinc transporter *SLC30A5*. Affected individuals shared a similar phenotype of cardiomyopathy and deceased perinatally.

## Materials and methods

### Family consolidation

Families were consolidated via the online match making platform GeneMatcher [12].

### Exome sequencing

ES was performed for seven of the eight available family members with the exception of the father of family 1, who was subjected to targeted Sanger sequencing for verification of the detected variant. After library preparation (Nextera DNA Flex, Illumina), target-enrichment and paired-end sequencing was performed differently for families 1 and 2. While the Human Core Exome, Twist Bioscience, and a NovaSeq 6000 Instrument (S1 Reagent Kit, 300 cycles, Illumina) were used for family 1; xGen^®^ Exome Research Panel v1.0, IDT, and a HiSeq instrument (SBS Kit v4, Illumina) were the methods used in case of family 2. For all seven ES, coverage of more than 20x was achieved in more than 95% of target sequences.

### Variant prioritization

For family 1, bioinformatic analysis was performed using the software Varfeed and Varvis (Limbus, Rostock, Germany). For family 2, bioinformatic analysis was performed using NextGENe (SoftGenetics, PA, USA). Mapping was carried out using hg19.

We evaluated all variants that are annotated in mutation databases (primarily HGMD and ClinVar [13, 14]) as well as rare (minor allele frequency below 1%) potential protein-influencing variants. We prioritized the variants based on minor allele frequency, inheritance mode, and potential predicted pathogenicity (based on a number of in silico predictions). Since no pathogenic or likely pathogenic variants could be identified in any known disease genes in either family, we continued evaluation of the sequencing data in a scientific setting on the basis of a previously written consent. Further variants in genes with no prior association to disorders have then been prioritized based on the mentioned parameters above as well as on attributes of the genes, like functional plausibility and gene constraints (i.e., LOEUF value and *Z*-score [15]). Additional aspects including availability of animal models, interaction partners, and plausibility of the symptoms were also considered in regard to the function of the gene.

## Results

Detailed clinical data can be found in Table 1.

**Table 1:** Detailed clinical information on individuals.

Patient index	1.1	1.2	1.3	2.1
Genetic data				
SLC30A5 variant (GRCh37; NM_022902.4)	c.832_836del p.(Ile278Phefs*33) chr5:g.68411799_68411803del			c.1981_1982del p.(His661Tyrfs*10) chr5:g.68419235_68419236del
Zygosity	Homozygous			Homozygous
General				
Consanguinity	Yes			Yes
Sex	Female	Male	Female	Male
Other genetic investigations	Karyotyping: 46,XX; gene panel sequencing	Karyotyping: 46,XY	Karyotyping: 46,XX	Array analysis unobtrusive, Karyotyping: 46,XY
Age at last assessment	30^+6^	27^+5^	31^+3^/32^+0^ (p.m.)	1st day of life
Pregnancy				
Gravidity and parity	G1P1	G2P2	G3P3	G2P2
Gestational parameters	Spontaneously occurring pregnancy, normal sonographic screenings at 11 and 20 weeks, gestational diabetes, Rh prophylaxis	Spontaneously occurring pregnancy, first sonographic screenings normal, Rh prophylaxis	Spontaneously occurring pregnancy, first sonographic screenings normal, Rh prophylaxis	Spontaneously occurring; no abnormalities until 31st week of gestation
Prenatal complications/birth history?	Generalized hydrops fetalis	22nd week of gestation: right ventricular hypertrophy, cardiomegaly, later on beginning hydrops fetalis	29^+5^ weeks of gestation: polyhydramnios, release punctures, hydrops fetalis, intrauterine fetal death, cardiomyopathy	31st week of gestation: polyhydramnios; 33rd week: hypertrophic right ventricle, congenital limb contractures
Decease	25 min after birth	4th day of life	IUFD after 31 gestational weeks	5th day of life
Birth mode	Emergency C-section due to pathologic CTG	C-section	Spontaneous	C-section
APGAR, umbel. Cord pH	01/01/01, 7.16	03/04/04, 7.34	n.a. (IUFD)	APGAR: 01/05/07
Amniotic fluid	Polyhydramnios, TORCH normal	Normal	Polyhydramnios	n.a.
Auxological data at birth				
Birth weight (SD)	1720 g (0.55)	1120 g (0.12)	1185 g (−1.54)	2770 g (−0.82)
Birth length (SD)	37 cm (−1.12)	36.5 cm (−0.11)	40 cm (−0.79)	n.a.
Birth OFC (SD)	32 cm (1.61)	28 cm (0.85)	29 cm (−1.69)	n.a.
Postmortem investigation				
Clinical	Massive hydrops fetalis, hypertrophic placenta, three umbilical cord vessels	Hydrops fetalis, immature placenta, three umbilical cord vessels	Hydrops fetalis, immature placenta, three umbilical cord vessels	Cystic neck hygroma
Autopsy	n.p.	Histological verification of non-compaction-type cardiomyopathy	n.p.	Neuropathological investigation: no pathological findings
X-ray	n.p.	Normal fetogram	n.p.	Small ribs
Other features				
Dysmorphic features	No	No	Small deep-set ears, flat facial profile	Mild dysmorphic features
Genitourinary anomalies	n.a.	No	n.a.	Hypoplastic scrotum
Cardiovascular anomalies	n.a.	Non-compaction-type cardiomyopathy, ventricular bradycardia, arrhythmia with intermittent 2:1 conduction of normal atrial frequency	Cardiomyopathy	Right ventricular hypertrophy, ventricular tachycardia, broad-complex tachycardias, and bradycardia
Neurological anomalies	n.a.	Immature gyration, frontal hygroma, intraventricular, and parenchymal hemorrhage beginning on 2nd day of life	No	No
Skin anomalies	Skin edema	Skin edema	Skin edema	Dystrophic nails
Family history	Apart from these 3 affected siblings family history negative for hydrops or CMP			Elder sister healthy, family history uneventful

### Clinical description

#### Family 1

All three pregnancies in family 1 were affected by complications with fetal hydrops and perinatal death. Postmortem examinations were performed on two of the three children and both were found to have cardiomyopathy. The healthy parents are first cousins.

**Fig. 1:**
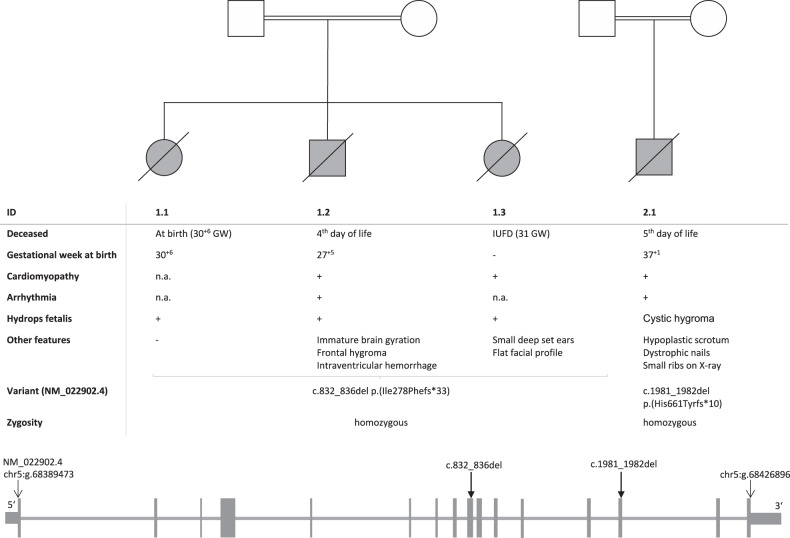
Overview on individuals. The figure lists key information on all affected individuals including variant postions. The conventional symbols were used for the pedigrees.

**Fig. 2:**
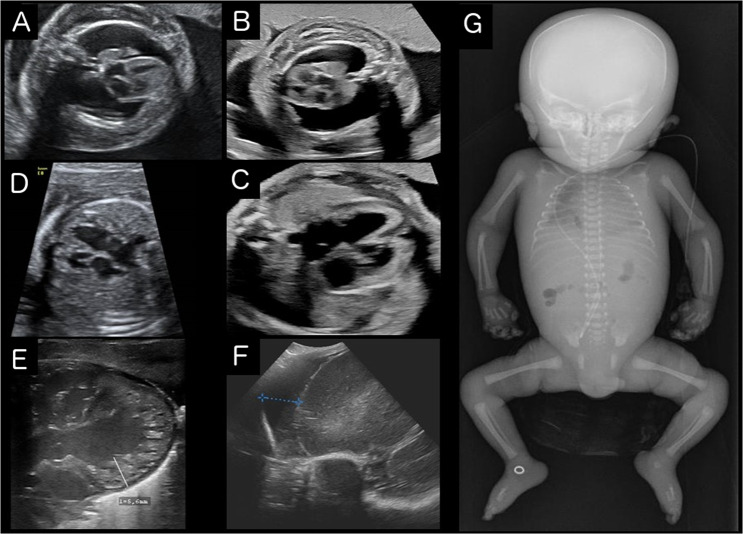
Imaging findings of the affected individuals. Prenatal ultrasound scans at the level of four-chamber view of individuals of family 1 (**A**: Voluson S8, AB2-7 convex abdominal ultrasound transducer 2–8 MHz; **B**: Voluson E8, RAB 6-D convex abdominal transducer 2–8 MHz; **C**, **D**: Philips EPIQ Elite, V 2–7 convex abdominal ultrasound transducer 2–9 MHz): fetus 1.1 at 28 weeks of gestation (**A**) and fetus 1.3 at 31 weeks of gestation (**B**) showing edematous skin and lungs maximally compressed by massive pleural effusions, polyhydramnios. Prenatal myocardial hypertrophy in fetus 1.2 at 25 weeks of gestation (**C**) and in fetus 1.3 at 28 weeks of gestation (**D**). Postnatal ultrasound scans of individual 1.2 (Zonare ZS3, Mindray, USA): **E** spongy left ventricular myocardium (nonstandard plane nonstandard with 20 MHz linear transducer), **F** frontal hygroma in parasagittal view (blue crosses mark a distance of 1.2 cm, 7,5 MHz curved array transducer). **G** Postmortem babygram of individual 1.2 (born 31^+2^, deceased on the 4th day of life) showing thickened soft tissue and pleural effusion on the right side, normal skeletal findings (color figure online).

The first pregnancy (Fig. 1, 1.1) was uneventful apart from gestational diabetes. Rhesus prophylaxis was carried out properly. Sonographic screenings at 11 and 20 weeks were inconspicuous. A checkup at 30^+6^ weeks of gestation showed a pathologic CTG and pathologic Doppler as well as a massive fetal hydrops (Fig. 2A). An emergency cesarean section was carried out immediately and the girl was born with 37 cm length (13 percentile, *Z* = −1.12 [16]), 1720 g weight (71 percentile, *Z* = 0.55), and 32 cm head circumference (95 percentile, *Z* = 1.61); APGAR 01/01/01, umbilical cord pH 7.16. Despite extensive resuscitation efforts, the infant girl remained bradycardic and deceased. Microarray analysis and gene panel sequencing were inconspicuous.

In the second pregnancy (Fig. 1, 1.2), a right ventricular hypertrophy and a cardiomegaly were diagnosed by ultrasonic imaging in the 22nd gestational week, but the amount of amniotic fluid remained normal at this time. As a consequence of a commencing hydrops fetalis that was observed in the 28th gestational week, an attempt was made to save the child by a cesarean section at 27^+5^ weeks (APGAR 03/04/04, umbilical cord pH 7.34). Birth measurements were normal for gestational age (length of 36.5 cm (46 percentile, *Z* = −0.11), weight of 1120 g (55 percentile, *Z* = 0.12), and head circumference of 28 cm (80 percentile, *Z* = 0.85)). ECG showed ventricular bradycardia with wide chamber complexes with 2 to 1 conduction of the normal atrial frequency, arrhythmia was refractory to therapy. Echocardiography suggested a non-compaction cardiomyopathy (Fig. 2E).

Brain sonography on the 1st day of life revealed an immature gyration and frontal hygroma (Fig. 2C). At age 2 days, pronounced thalamic and intraventricular hemorrhage was observed. Diagnostic tests did not show any evidence of metabolic disease or infections.

Despite intensive care, the boy died after 4 days. Postmortem examination did not show evidence of congenital malformations. Histological examination of cardiac tissue revealed the suspected cardiomyopathy of the non-compaction type. Furthermore, an immature placenta and a normal fetogram on X-ray were found.

Subsequent ES of the mother, child 1.1 and child 1.2 did not reveal robust variants that could explain the phenotype in question.

The third pregnancy (Fig. 1, 1.3) showed a similar course. Polyhydramnios, mild myocardial hypertrophy, and small ventricular septal defect were diagnosed in the 28th week of gestation; hydrops was seen a few days later (Fig. 2B–F). Amniotic fluid was punctured twice (at 29th and 30th gestational weeks) to relieve the polyhydramnios. Chromosome analysis was normal (46,XX). Intrauterine fetal death (IUFD) of the female fetus (Fig. 1, 1.3) was diagnosed at gestational week 31^+3^. Clinical examination of the deceased fetus after birth at week 32^+0^ showed hydrops fetalis and normal measurements (length 40 cm (21 percentile, *Z* = −0.79), weight of 1185 g (6 percentile, *Z* = −1.54), head circumference of 29 cm (34 percentile, *Z* = −1.69)). The fetus had small deep-set ears, a flat facial profile, and normal hands and feet.

We performed exome analysis on all three affected siblings.

#### Family 2

The boy (Fig. 1, 2.1) was the second child of consanguineous parents with an uneventful family history. During the complicated pregnancy, polyhydramnios was diagnosed at 31st week and a right ventricle hypertrophy in the course of the 33rd week. Birth was carried out per cesarean section at gestational week 37^+1^ (APGAR 01/05/07) and the birth weight was 2770 g (21 percentile, *Z* = −0.82). The child was intubated immediately after birth due to suspected heart disease. Thereafter, cardiac arrhythmias with persistent ventricular tachycardia occurred and turned out to be refractory to therapy. Cardiopulmonary resuscitation was performed three times without success after the occurrence of ventricular tachycardia and bradycardia leading to asystole. Peripheral Venous Arterial ECMO was initiated because of insufficient circulation. Due to persistent arrhythmias and severe right ventricle insufficiency, all life-saving measures were discontinued. As a consequence of broad-complex ventricular tachycardia, the child died at the age of 5 days.

Physical examination revealed mild dysmorphic features, a cystic neck hygroma and a hypoplastic scrotum, dystrophic nails, and small ribs on X-ray. The neuropathological investigation of the right quadriceps femoris showed age-appropriate skeletal muscles and no pathological findings.

### Genetic results

In family 1, ES revealed the homozygous frameshift variant NM_022902.4(SLC30A5):c.832_836del p.(Ile278Phefs*33) in all three fetuses. Both parents are heterozygous. In family two, ES affirmed the homozygous frameshift variant NM_022902.4(SLC30A5):c.1981_1982del p.(His661Tyrfs*10) in the affected child. Both parents are heterozygous.

Both detected variants are absent from publicly available databases, including gnomAD [15], and also from our in-house databases in Leipzig and Zürich. We assume that both frameshifts probably result in a full LoF of the protein. In gnomAD, no homozygous LoF variants can be found in this gene.

## Discussion

Here we describe four individuals in two unrelated consanguineous families with lethal cardiomyopathy, arrhythmia, and hydrops fetalis/cystic hygroma in whom we identified homozygous variants in *SLC30A5*.

We identified the homozygous variant NM_022902.4:c.832_836del p.(Ile278Phefs*33) in all three affected individuals of the first family, while the affected child of the second family carried the homozygous variant NM_022902.4:c.1981_1982del p.(His661Tyrfs*10). We assume that the position of the variants in or outside of any of the protein domains is irrelevant as both variants lead to frameshifts and are predicted to result in a LoF due to nonsense mediated decay. The minor allele frequency of all *SLC30A5* LoF variants in gnomAD is 1:4330 with no reported homozygous LoF variants. Even assuming that the first identified LoF in this study is not relevant to the phenotype, the probability of identifying a second homozygous LoF in *SLC30A5* in a cohort of 50,000 cases (there are 44,000 entries in GeneMatcher) by chance is 0.0027, making a coincidental event highly improbable.

In addition, all four affected patients show a strong phenotypic similarity including perinatal lethality and either fetal hydrops or cystic hygroma. Apart from the first affected child (1.1), who did not receive echocardiography, all other individuals had cardiomyopathy. Both children that were born alive and later received clinical examination (1.2 and 2.1) were diagnosed with arrhythmia. The other two children died of an unknown cause; arrhythmia would fit into the clinical course. We therefore assume these variants to be causative for the clinical features.

*SLC30A5* encodes for ZnT-5, a zinc transporter. Zinc is the most abundant trace element in human cells [17] and about 10% of all eukaryotic proteins need zinc to fulfill their cellular activity [18]. This makes zinc essential for a broad spectrum of cellular functions [19], e.g., transcription, ATP production and mitochondrial apoptogenesis [20], activation of enzymes in the early secretory pathway [21], microtubule formation [22], and intracellular signaling [23]. The intracellular zinc homeostasis is hence kept within narrow limits by a battery of zinc transporters [24]; mainly 14 zinc importers (*SLC39* family, ZIP channels) and 10 Zn exporters (*SLC30* family, ZnT channels). Of the 10 ZnTs, ZnT-10 (*SLC30A10*, OMIM *611146, OMIM #613280, [25]) is associated with the autosomal recessive hypermanganesemia with dystonia 1, while ZnT-2 (*SLC30A2*, OMIM *609617, OMIM #608118, [26]), ZnT-5 (*SLC30A5*, OMIM *607819, [27]), and ZnT-6 (*SLC30A5*, OMIM *611148, [27]) have been linked to a reduced amount of zinc in mother milk leading to dermal symptoms in the infant. Of the 14 ZIPs, four are associated with autosomal recessive disorders including a broad spectrum of phenotypes: acrodermatitis enteropathica (ZIP4, *SLC39A4*, OMIM *607059, OMIM #201100, [28]), congenital disorder of glycosylation type IIn with intellectual disability and short stature (ZIP8, *SLC39A8*, OMIM *607059 #616721, [29]), Ehlers–Danlos syndrome of spondyloplastic type 3 (ZIP13, *SLC39A13*, OMIM *608735 #612350, [30]), hypermanganesemia with dystonia 2 (ZIP14, *SLC39A14*, OMIM *608736 #617013, [31]), and one autosomal dominant phenotype of severe myopia (ZIP5, *SLC39A5*, OMIM *608730 #615946, [32]). In addition, several studies have shown variable clinical presentation of loss of zinc transporters in mice [33, 34]. The broad spectrum of both clinical presentations and pathomechanisms is in line with the large number of cellular functions of zinc. The limitation of symptoms to individual organs could be explained by specific expressions of cellular functions through different zinc transporters. ZIP4, for example, is associated with acrodermatitis enteropathica and mostly expressed in the digestive tract.

*SLC30A5* encodes ZnT-5, which is localized at membranes of endoplasmic reticulum and Golgi apparatus. It forms heterodimers with ZnT-6 [21]. Its function is the influx of Zn^2+^ ions into the lumen of the cell organelles. With respect to the various functions of zinc, it is conceivable that a disturbed zinc homeostasis due to a total LoF of ZnT-5 could have remarkable consequences.

Inoue et al. [34] have examined ZnT-5, the Zn^2+^ channel encoded by *SLC30A5* in a knockout mouse model. The mice showed reduced body growth and reduced bone density. About 60% of the mice died due to bradyarrhythmia. As both knockout mice and affected individuals had cardiac arrhythmia, we see an analogy of the phenotypic spectrum. We consider the results of the study of Inoue et al. as a further line of evidence regarding the association between bi-allelic LoF in *SLC30A5* and cardiomyopathy. We also suggest that the pathomechanism is possibly a disturbed cardiac conduction. This is further supported by the results of Lin et al. [33], who showed that *SLC39A8*-null mice exhibit cardiomyopathic phenotypes of the non-compaction type, which was also present in at least two of the four affected patients. ZIP8 (*SLC39A8*) is one of several Zn^2+^ transporters that are responsible for the influx of Zn^2+^ into the cell. This is a requirement for the subsequent transport of the ions into the endoplasmic reticulum by ZnT-5. It seems therefore plausible that a LoF of ZnT-5 can lead to a similar phenotype as the loss of ZIP8. However, the specific pathomechanism remains unknown up until now and needs to be investigated in future studies. Additional families with affected children will help to further statistically substantiate the suspected association.

Interestingly, in all studied cases hydrops fetalis/cystic hygroma occurred late in pregnancy, with a diagnosis being made as late as 20 weeks of gestation or even at birth, albeit regular sonographic controls. McCoy et al. [35] even state that a hydrops fetalis is more likely to be caused by cardiothoracic abnormalities the later in the pregnancy the diagnosis was made. Cystic hygroma normally develops by the end of the 6th week of gestation but was not found for individual 2.1 on sonographic screenings before birth.

In conclusion, here we describe four individuals from two families with perinatal lethal cardiomyopathy, all of which have bi-allelic LoF variants in the zinc transporter *SLC30A5*. For the first time, we are able to link bi-allelic LoF variants in this gene with a human disease. Although the pathomechanisms are not yet understood, they seem to fit in the spectrum of zinc transporters. Further analyses are needed to investigate these mechanisms.
